# Breathing patterns in people with exercise‐induced laryngeal obstruction

**DOI:** 10.14814/phy2.15086

**Published:** 2021-11-25

**Authors:** Astrid Haugen Lie, Ingvild Grønnevik, Bente Frisk, Ola Drange Røksund, Ida Hammer, Maria Vollsæter, Thomas Halvorsen, Hege H. Clemm

**Affiliations:** ^1^ Department of Clinical Science University of Bergen Bergen Norway; ^2^ Department of Health and Functioning Western Norway University of Applied Sciences Bergen Norway; ^3^ Department of Pediatric and Adolescent Medicine Haukeland University Hospital Bergen Norway

**Keywords:** EILO, exercise physiology, laryngeal obstruction, respiratory physiology

## Abstract

Exercise‐induced laryngeal obstruction (EILO) is common, but we lack readily available diagnostic tools. The larynx represents an important point of resistance in the airways, and we therefore hypothesized that EILO is associated with characteristic breathing patterns possible to record from a standard incremental ergospirometry test. We studied 24 individuals with moderate/severe EILO and 20 individuals with no‐EILO, mean (SD) age 17 (6.1) and 24 (6.4) years, respectively. EILO versus no‐EILO was verified from maximal continuous laryngoscopy treadmill exercise (CLE) tests, which also included ergospirometry. We described the relationships between minute ventilation (${\dot{V}}_{E}$) versus tidal volume (*V*_T_) and ${\dot{V}}_{E}$ versus carbon dioxide output ($\dot{V}{\text{CO}}_{2}$), using respectively quadratic and linear equations, and applied adjusted regression models to compare ergospirometry data and curve parameters. Compared to the no‐EILO group, the group with EILO had prolonged inspiratory time (*T*_in_), lower breathing frequency (*B*_f_), lower ${\dot{V}}_{E}$, and lower inspiratory flow rate (${\dot{V}}_{\text{in}}$) at peak exercise. Mathematical modeling of the breathing pattern relationships was feasible in both groups, with similar coefficients of variation. For ${\dot{V}}_{E}$ versus *V*_T_, the mathematical curve parameters were similar. For ${\dot{V}}_{E}$ versus $\dot{V}{\text{CO}}_{2}$, the slope was similar but the intercept was lower in the EILO group. EILO was associated with prolonged *T*_in_, lower *B*_f_, ${\dot{V}}_{E}$, and ${\dot{V}}_{E}$. The relationship between ${\dot{V}}_{E}$ versus *V*_T_ was similar, whereas for ${\dot{V}}_{E}$ versus $\dot{V}{\text{CO}}_{2}$, the slope was almost parallel but shifted downward for the EILO group. Most ergospirometry data overlapped, except ${\dot{V}}_{\text{in}}$ which discriminated between EILO and no‐EILO in a promising way.

## Graphical abstract

EILO was associated with prolonged *T*_in_, lower *B*_f_, ${\dot{V}}_{E}$, and ${\dot{V}}_{\text{in}}$. The relationship between ${\dot{V}}_{E}$ versus *V*_T_ was similar, whereas for ${\dot{V}}_{E}$ versus $\dot{V}{\text{CO}}_{2}$. Most ergospirometry data overlapped, except ${\dot{V}}_{\text{in}}$ which discriminated between EILO and no‐EILO in a promising way.

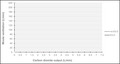

## 1 INTRODUCTION

Exertional dyspnea is common and can be caused by a variety of conditions in multiple organ systems (Abu‐Hasan et al., 2005). Symptom descriptions often overlap across etiologies and cannot readily be used to distinguish between diseases (Abu‐Hasan et al., 2005). Diagnosing respiratory complaints solely from patient‐reported symptoms may therefore lead to mistakes (Hallstrand et al., 2002; Parsons et al., 2007), and guidelines strongly advocate objective diagnostic work‐up (Parsons et al., 2013; Pavord et al., 2017). Within respiratory causal pathways, investigations tend to focus on diseases involving bronchial pathology, such as exercise‐induced asthma (EIA). However, it is becoming increasingly clear that the larynx is heavily involved in exertional dyspnea, both as an independent source of airflow obstruction (Christensen et al., 2015; Clemm et al., 2018; Halvorsen et al., 2017; Johansson et al., 2015), and perhaps as a contributing part of asthma (Low et al., 2011, 2017) and COPD (Baz et al., 2015).

Objective evaluation for EIA is standardized and widely performed, whereas the evaluation of exercise‐induced laryngeal obstruction (EILO) still tends to rest on symptom descriptions from patients. A Task Force statement from 2013 suggests that EILO should be diagnosed by continuous laryngoscopy exercise testing (CLE test), preferably performed throughout a maximal cardiopulmonary exercise test (CPET). The CLE test requires skilled clinicians and technicians as well as advanced equipment. Such resources are not widely available, thus less invasive ways of diagnosing EILO are clearly an unmet need.

The larynx represents the single most important point of resistance of the airway tree, accounting for approximately 25% of total airway resistance at rest and more during exercise (Baier et al., 1977; Ferris et al., 1964). EILO may therefore likely lead to alter and perhaps characteristic patterns of breathing during an exercise session. We hypothesized that changes in breathing patterns can be detected in parameters provided by a standard CPET, which is routinely performed worldwide. We also considered the reverse sequence of events, that is, whether an inappropriate breathing pattern may cause EILO. In either case, if breathing patterns in EILO differ in characteristic ways, such idiosyncrasies could be utilized for diagnostic purposes. To explore these hypotheses, we aimed to test if mathematical models (linear and quadratic equations), could be used to describe the breathing patterns obtained from CPETs in patients with EILO, and if the curve parameters in the regression models differed between a group with EILO and a no‐EILO control group.

## 2 METHODS

### 2.1 Subjects

This was an explorative cross‐sectional study based on two different groups of young people: patients with moderate to severe EILO randomly selected from the EILO register at Haukeland University Hospital in Bergen, Norway. This group was compared to a sample of highly active young people with no exercise‐related respiratory complaints who had performed a CLE test without visual signs of laryngeal obstruction.

The Committee on Medical Research Ethics of Western Norway approved this study (REK 2016/1898 and REK 2014/601) and informed written consents were obtained from all participants.

### 2.2 Spirometry, CPET, and CLE

Spirometry was performed using a Vyntus^®^ PNEUMO spirometer (Vyaire Health Care) according to standard guidelines (Graham et al., 2019), recording forced vital capacity (FVC) and forced expiratory volume in 1 s (FEV_1_) reported as *z*‐scores adjusted for sex, height, and age (Quanjer et al., 2012).

The CLE test was performed as previously described by Heimdal et al. (2006). Briefly, a transnasal flexible fiberoptic laryngoscope (Olympus ENF‐P3©) with diameter 3.5 mm was introduced after applying a decongestive nasal spray (Rhinox©) and local anesthesia (Xylocaine©) and secured using a custom designed helmet in a position allowing for a good view of the larynx. A facemask (Hans Rudolph Inc.) connected the patient to a Vyntus SentrySuite CPET unit (Vyaire Health Care). An incremental treadmill test (Woodway PPS 55 Med) was applied, using a pre‐set modified Bruce protocol identical for all participants. Treadmill speed and elevation were gradually increased every 60 s from an initial slow walking phase. After baseline was established, subjects ran to exhaustion (Cumming et al., 1978). An external camera and a sound recorder documented the patient running on the treadmill and the breath sounds. The test was considered successful when the subject indicated exhaustion, preferably supported by a plateau in oxygen consumption ($\dot{V}O_{2}$) and/or the heart rate (HR) or stopped running due to respiratory distress. The test duration and completed treadmill distance were recorded.

Oxygen consumption $\dot{V}O_{2}$, carbon dioxide output ($\dot{V}{\text{CO}}_{2}$), tidal volume (*V*_T_), breathing frequency (*B*_f_), inspiratory time (*T*_in_), expiratory time (*T*_ex_), and HR were measured directly, while minute ventilation (${\dot{V}}_{E}$) was calculated from *V*_T_ and *B*_f_, and inspiratory flow rate (${\dot{V}}_{\text{in}}$) from *V*_Tin_/*T*_in_. The video recordings of the larynx, the external film, the soundtracks, and the CPET data were all coordinated in time and stored in one common file for later assessment.

Laryngeal movements during the test were scored retrospectively from the video recordings by two experienced raters (HHC and ODR), using the CLE score system (Christensen et al., 2015; Maat et al., 2009). A score of 2–3 at either the glottic or supraglottic level at peak exercise was required for inclusion as an EILO patient, whereas scores of 0 at both laryngeal levels were required to participate as no‐EILO.

### 2.3 Data processing and statistics

Breath‐by‐breath measurements were averaged over 20 s intervals. Baseline variables were compared using independent samples *t*‐tests, and linear regressions were used to adjust for group differences regarding sex, height, and age when relevant.

Each participant's breathing pattern was modeled by the relationships ${\dot{V}}_{E}$ versus *V*_T_ and $\dot{V}{\text{CO}}_{2}$ versus ${\dot{V}}_{E}$ using a quadratic model (*V*_T_ = *a* + *b*·${\dot{V}}_{E}$ + *c*·${\dot{V}}_{E}^{2}$) and a linear model (${\dot{V}}_{E}$ = *a* + *b*·$\dot{V}{\text{CO}}_{2}$ up to the point of isocapnic compensation), respectively. F‐statistic was used to calculate the goodness of fit for each model. All models were subsequently evaluated separately by three factors: the adjusted coefficient of determination (adjusted *R* ^2^), *p*‐value <0.05, and visual curve suitability. A visual fitness and a *p*‐value <0.05 were required for inclusion in further analyses.

The dependent variables in the linear model, the intercept (a) and the slope (b), and additionally in the quadratic model the curvature (c), were analyzed by bivariate and multivariate linear regression models. EILO versus no‐EILO, age, sex, height, *T*_in_, and FEV_1_ were potential explanatory variables in both models. FEV_1_ was included as absolute values, and not as z‐scores as sex, age, and height were already included in the regression models. FVC was not included in the analyses due to co‐linearity with FEV_1_. Variables to be included in the multivariate linear regression models were selected based on backward stepwise regression with *p* < 0.2 as cut‐off for inclusion. The variables EILO versus no‐EILO and sex were included a priori.

Estimated regression coefficients are presented with 95% confidence intervals (CIs) and *p*‐values. The two‐sided significance level was set at 0.05. The data analyses were performed using IBM SPSS Statistics 25 (SPSS Inc.).

## 3 RESULTS

Subject characteristics, resting pulmonary function, and peak responses to the CPET are summarized in Table 1. All participants ran to exhaustion with at least 95% of maximum HR. Baseline characteristics differed between the EILO versus no‐EILO groups regarding age, sex, and height. Adjusted for these differences, the two groups differed regarding $\dot{V}O_{2\text{peak}}$, *B*_f_, ${\dot{V}}_{E}$, *T*_in_, and ${\dot{V}}_{\text{in}}$. The EILO group had lower z‐FVC and z‐FEV_1_, but the ratio FEV_1_/FVC was within normal range for all participants.

**TABLE 1:** Participant characteristics and peak responses to progressively incremental exercise test on treadmill

	EILO *n* = 24	No‐EILO *n* = 20	*p*‐values unadjusted	*p*‐values adjusted
Male/female	2/22	9/11		
Age (years)	17.0 ± 6.1	24.3 ± 6.4	**<0.001**	NA
Weight (kg)	60.9 ± 11.3	67.0 ± 11.6	0.083	NA
Height (cm)	164.9 ± 6.1	175.5 ± 12.1	**0.001**	NA
Mean CLE scores at peak, glottic/supraglottic	2.57/2.35	0/0	NA	NA
FVC (L)	3.78 ± 0.51	5.10 ± 1.21	**<0.001**	0.106
FVC *z*‐score	−0.56 ± 0.46	0.62 ± 1.08	**<0.001**	NA
FEV_1_ (L)	3.26 ± 0.52	4.12 ± 0.88	**0.001**	0.312
FEV_1_ *z*‐score	−0.50 ± 0.62	0.55 ± 1.06	**0.001**	NA
FEV_1_/FVC	0.86 ± 0.04	0.82 ± 0.07	**0.019**	0.369
HR_peak_ (min^−1^)	186 ± 19	184 ± 11	0.676	0.650
$\dot{V}O_{2\text{peak}}$(mlˑmin^−1^)	2697 ± 336	3622.15 ± 818	**<0.001**	**0.039**
$\dot{V}{\text{CO}}_{2\text{peak}}$(mlˑmin^−1^)	3234 ± 553	4372.20 ± 945	**<0.001**	0.061
RER	1.19 ± 0.08	1.21 ± 0.013	0.468	0.726
${\dot{V}}_{E\text{peak}}$(Lˑmin^−1^)	90.8 ± 17.5	133.35 ± 31.3	**<0.001**	**0.005**
*B*_f_ (min^−1^)	45 ± 8	51 ± 9	**0.030**	**0.030**
*V*_T_ (L/breath)	2.05 ± 0.36	2.67 ± 0.63	**<0.001**	0.406
*T*_in_ (s)	0.70 ± 0.14	0.60 ± 0.11	**0.014**	**0.040**
*T*_ex_ (s)	0.69 ± 0.15	0.62 ± 0.12	0.109	0.071
*T*_in_/*T*_tot_ (s)	0.50 ± 0.03	0.49 ± 0.03	0.199	0.750
${\dot{V}}_{\text{in}}$(L/min)	181.57 ± 35.95	274.33 ± 67.99	**<0.001**	**0.005**

Data are presented as mean ± 1 standard deviation. *p*‐values denote group differences unadjusted or adjusted for sex, age, and height. Continuous laryngoscopy exercise (CLE) test scores were rated at peak exercise at glottic and glottic levels.

Bold indicates *p*‐value < 0.05.

Abbreviations: ${\dot{V}}_{\text{in}}$, inspiratory flow rate (*V*_Tin_/*T*_in_); $\dot{V}{\text{CO}}_{2\text{peak}}$, peak carbon dioxide output; ${\dot{V}}_{E\text{peak}}$, peak minute ventilation; $\dot{V}O_{2\text{peak}}$, peak oxygen uptake; *B*_f_, breathing frequency; FEV_1_, forced expired volume in 1 s; FVC, forced vital capacity; HR_peak_, peak heart rate; NA, not applicable; RER, respiratory exchange ratio; *T*_ex_, expiratory time; *T*_in_/*T*_tot_, relationship between ratio of mean inspiratory time to total time of respiratory cycle; *T*_in_, inspiratory time; *V*_T_, tidal volume.

Table 2 reports results from the regression models used to test the associations between potential explanatory variables (including the grouping variable EILO vs. no‐EILO) versus relevant ventilatory parameters. In the adjusted analyses, EILO was associated with prolonged *T*_in_ and *T*_ex_, a lower B_f_, and lower ${\dot{V}}_{\text{in}}$. In addition to EILO, FEV_1_ and sex contributed to both ${\dot{V}}_{E}$ and ${\dot{V}}_{\text{in}}$. EILO did not contribute to *V*_T_ and *T*_in_/*T*_tot_. *V*_T_ was mainly explained by FEV_1_ and sex, without contribution from EILO.

**TABLE 2:** Regression models testing associations between potential explanatory variables (including EILO versus no‐EILO) versus relevant ventilatory parameters

	Bivariate		Multivariate		
	*B*	*p*‐value	Std. *B*	95% CI	*p*‐value
*V*_t_ (L)					
EILO versus no‐EILO	0.981	**<0.001**	−0.090	−0.309, 0.096	0.293
Sex	0.981	**<0.001**	0.210	0.006, 0.562	**0.046**
Age	0.036	**0.003**			
Height	0.044	**<0.001**			
FEV_1_	0.642	**<0.001**	0.686	0.339, 0.660	**<0.001**
*B*_f_ (breaths/min)					
EILO versus no‐EILO	−5.810	**0.030**	−0.427	−13.626, −1.540	**0.015**
Sex	2.112	0.504	0.173	−5.184, 12.241	0.418
Age	0.166	0.388			
Height	−0.005	0.971	−0.345	−0.666, 0.086	0.127
FEV_1_	−0.027	0.987			
${\dot{V}}_{E}$(L/min)					
EILO versus no‐EILO	−42.558	**<0.001**	−0.331	–34.050, −8.958	**0.001**
Sex	53.636	**<0.001**	3.83	6.275, 40.751	**0.009**
Age	2.097	**0.001**			
Height	2.100	**<0.001**			
FEV_1_	31.117	**<0.001**	3.18	5.277, 25.213	**0.004**
*T*_in_ (sec)					
EILO versus no‐EILO	0.100	**0.014**	0.476	0.036, 0.221	**0.008**
Sex	−0.055	0.250	−0.271	−0.211, 0.045	0.196
Age	–0.004	0.150			
Height	–0.001	0.695			
FEV_1_	–0.005	0.835	0.410	−0.006, 0.142	0.071
*T*_ex_ (sec)					
EILO versus no‐EILO	0.068	0.109	0.363	0.005, 0.197	**0.039**
Sex	−0.023	0.638	−0.230	−0.212, 0.064	0.287
Age	−0.002	0.547			
Height	0.001	0.603	0.429	0.0003, 0.012	0.062
FEV_1_	0.006	0.812			
*T*_in_/*T*_tot_					
EILO versus no‐EILO	0.012	0.199	0.217	−0.009, 0.036	0.233
Sex	−0.009	0.426	−0.010	−0.034, 0.032	0.964
Age	−0.001	0.146			
Height	−0.001	0.136	−0.607	−0.004, 0.00004	0.055
FEV_1_	−0.003	0.602	0.557	−0.003, 0.046	0.080
${\dot{V}}_{\text{in}}$(L/min)					
EILO versus no‐EILO	−92.76	**<0.001**	−43.95	−73.836, −14.071	**0.005**
Sex	115.18	**<0.001**	59.53	21.112, 97.957	**0.003**
Age	4.67	**<0.001**	1.25	−0.684, 3.192	0.198
Height	4.73	**<0.001**			
FEV_1_	65.04	**<0.001**	23.73	1.130, 46.330	**0.040**

Bold indicates *p*‐value < 0.05.

Abbreviations: ${\dot{V}}_{\text{in}}$, inspiratory flow rate (*V*_Tin_/*T*_in_); *B*, unstandardized beta; *B*_f_, breathing frequency; *B*_f_, breathing frequency; CI, confidence interval; FEV_1_, forced expired volume in 1 s; Std. *B*, standardized beta; *T*_ex_, expiration time; *T*_in_/*T*_tot_, ratio of mean inspiratory time to total time of respiratory cycle; *T*_in_, inspiration time; *V*_E_, minute ventilation; *V*_T_, tidal volume.

**FIGURE 1:**
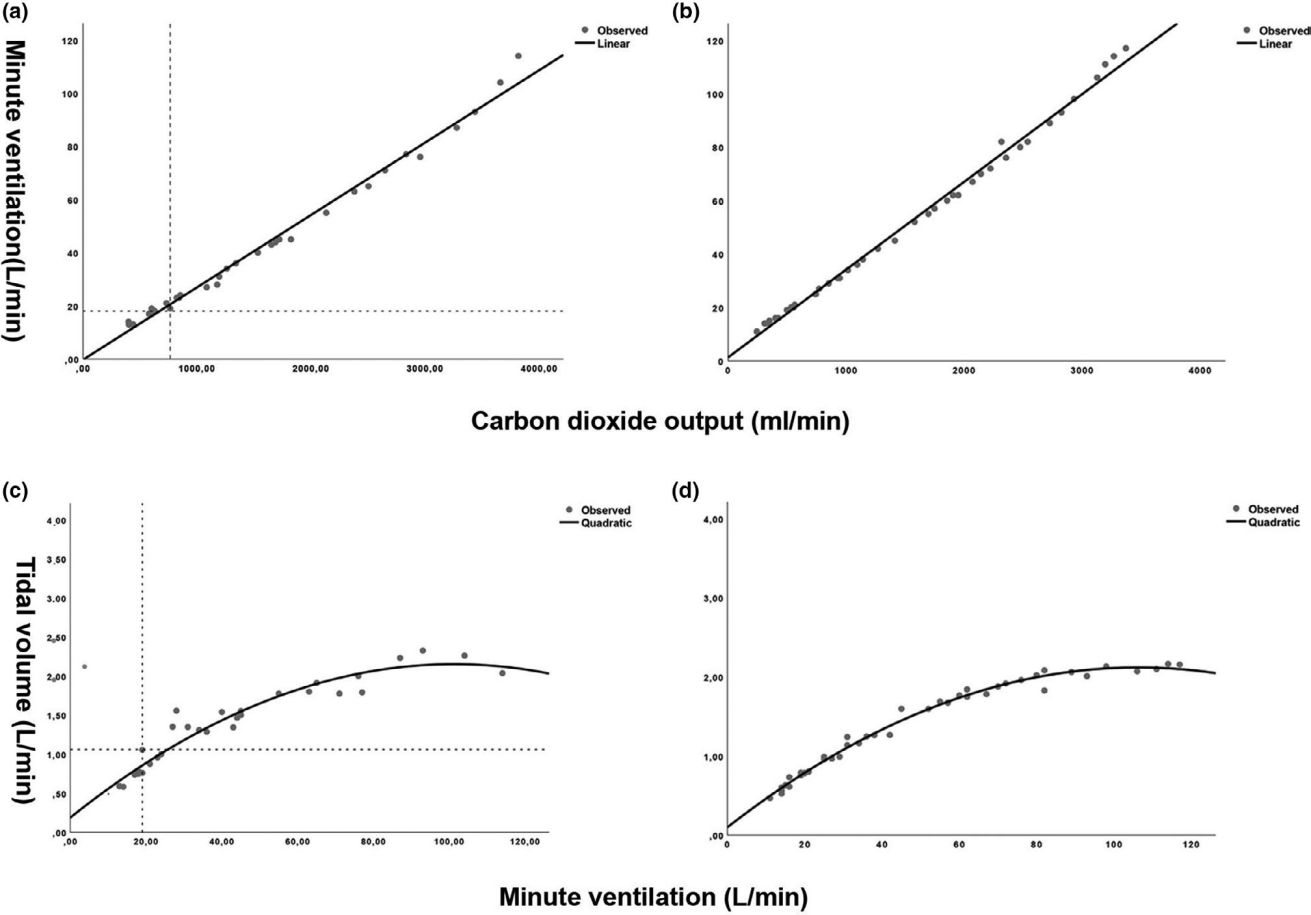
The relationships between minute ventilation (${\dot{V}}_{E}$) versus $\dot{V}{\text{CO}}_{2}$ for EILO (a) and no‐EILO (b) and tidal volume (VT) versus V̇E for EILO (c) and no‐EILO (d), respectively

**FIGURE 2:**
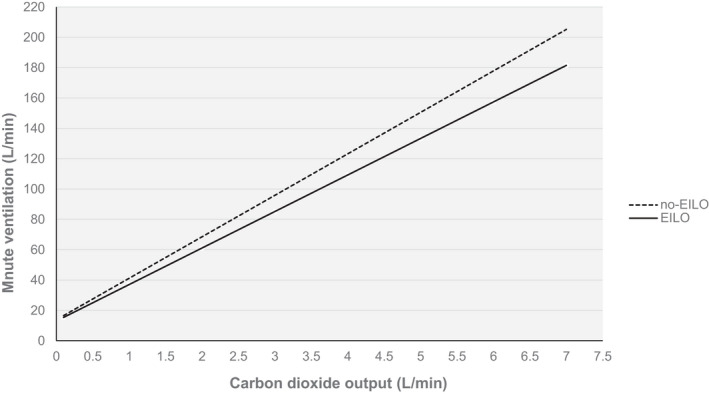
The relationships between ventilation and carbon dioxide output in the EILO and no‐EILO groups estimated by the regression equations

The relationship between ${\dot{V}}_{E}$ and $\dot{V}{\text{CO}}_{2}$ was satisfactorily described by a linear model (Figure 1), with *p*‐values <0.001 and mean *R^2^* of 0.986 (range 0.93–1.00) for all participants in both groups. The slope (curve parameter *b*) was similar in the EILO and no‐EILO groups, but the intercept (curve parameter *a*) was significantly related to EILO (lower) and *T*_in_ (higher), but not the other variables included in the model (Table 3). Thus, in the adjusted analysis, the ${\dot{V}}_{E}$ versus $\dot{V}{\text{CO}}_{2}$ slope was almost parallel in the EILO and no‐EILO groups, but with downward shift for the EILO group. The prediction equations for both groups are demonstrated in Figure 2.

**TABLE 3:** Multiple regression analyses for the curve parameters describing the relationship between ${\dot{V}}_{E}$ and $\dot{V}{\text{CO}}_{2}$ assuming a linear relationship (${\dot{V}}_{E}$ = *a* + b·$\dot{V}{\text{CO}}_{2}$)

	Bivariate		Multivariate		
	*B*	*p*‐value	Std. *B*	95% CI	*p*‐value
Curve parameter *a*					
EILO/no‐EILO	–0.468	0.622	–0.380	–4.606, –0.142	**0.038**
Sex	–1.630	0.131	–0.365	–5.579, 0.394	0.087
Age	0.039	0.552			
Height	0.012	0.797	0.539	–0.006, 0.320	0.058
FEV_1_	–0.288	0.635	–0.461	–3.950, 0.437	0.111
*T*_in_	8.099	**0.016**	0.452	3.286, 17.609	**0.005**
Curve parameter *b*					
EILO/no‐EILO	–0.001	0.207	–0.022	–0.002, 0.003	0.806
Sex	0.001	0.316			
Age	<0.001	0.545			
Height	<0.001	0.650	–0.310	<0.001, <0.001	0.109
FEV_1_	<0.001	0.876	–0.461	–0.001, 0.004	0.154
*T*_in_	–0.017	**<0.001**	0.700	–0.026, –0.011	**<0.001**

Bold indicates *p*‐value < 0.05.

Abbreviations: *B*, unstandardized beta; CI, confidence interval; FEV_1_, forced expired volume in 1 s; Std. *B*, standardized beta; *T*_in_, inspiration time.

The relationship between ${\dot{V}}_{E}$ and *V*_T_ was satisfactorily described by a quadratic model (Figure 1), with *p*‐values <0.001 and mean *R* ^2^ 0.916 (range 0.62–0.99) for all participants in both groups. All but five subjects with EILO had *R* ^2^ >0.84, and these five all had *p*‐values <0.001 and a visually fitted curve and were therefore included in further analyses. Group comparisons (EILO vs. no‐EILO) revealed no significant differences in intercept (a), slope (b), or curvature (c). The only variable that significantly influenced curve parameters *a* and *b* was *T*_in_ (Table 4).

**TABLE 4:** Bivariate and multivariate regression analyses for the curve parameters *a*, *b*, and *c*, describing the relationship between ${\dot{V}}_{E}$ and *V*_T,_ assuming a quadratic relationship; *V*_T_ = *a* + *b*·${\dot{V}}_{E}$ + *c*·${\dot{V}}_{E}^{2}$

	Bivariate		Multivariate		
	*B*	*p*‐value	Std. *B*	95% CI	*p*‐value
Curve parameter *a*					
EILO versus no‐EILO	–0.039	0.681	0.228	−0.083, 0.298	0.259
Sex	0.146	0.180			
Age	–0.002	0.754	−0.323	−0.022, 0.001	0.067
Height	0.001	0.781			
FEV_1_	0.022	0.634	0.311	−0.015, 0.195	0.090
*T*_in_	−0.596	0.084	0.311	−0.015, 0.195	**0.026**
Curve parameter *b* (10^−2^)					
EILO versus no‐EILO	0.06	0.935	−0.129	−0.20, 0.008	0.416
Sex	−0.003	0.655			
Age	0.01	0.779			
Height	0.02	0.488			
FEV_1_	0.37	0.123			
*T*_in_	0.054	0.025	0.384	0.011, 0.113	**0.019**
Curve parameter *c* (10^−4^)					
EILO versus no‐EILO	–0.74	0.313	0.199	−0.00004, 0.0001	0.302
Sex	0.011	0.168	0.344	−0.00003, 0.000214	0.134
Age	0.04	0.448			
Height	0.02	0.536	−0.492	−0.00001, 0.000001	0.107
FEV_1_	0.21	0.358	0.415	−0.00003, 0.0002	0.182
*T*_in_	−0.056	0.035	−0.331	−0.001, 0.000003	0.053

Note

The curve parameter *a* represents the intercept, the curve parameter *b* represents the slope, and the curve parameter *c* represents the curvature of the slope.

Bold indicates *p*‐value < 0.05.

Abbreviations: *B*, unstandardized beta; CI, confidence interval; FEV_1_, forced expired volume in 1 s; Std. *B*, standardized beta; *T*_in_, inspiration time.

The coefficient of variation for the estimate of *T*_in_/*T*_tot_ over the range of *V*_T_ was approximately 12% in both the EILO and no‐EILO groups.

The coefficient of variation for the estimate of ${\dot{V}}_{\text{in}}$ was higher, however similar in both groups with approximately 63% in the EILO group and 60% in the no‐EILO group.

## 4 DISCUSSION

This is the first study to compare breathing patterns obtained from maximal cardiopulmonary exercise tests in participants with and without laryngoscopically confirmed EILO. Commonly applied mathematical models could satisfactorily be utilized to describe cardiopulmonary exercise data in all participants. Compared to the no‐EILO group, EILO was associated with prolonged *T*_in_, lower B_f_, lower ${\dot{V}}_{E}$, and lower ${\dot{V}}_{\text{in}}$ at peak exercise. For the relationship between ${\dot{V}}_{E}$ versus *V*_T_, the curve parameters did not differ between the two groups. The coefficient of variation for *T*_in_/*T*_tot_ over the full range of ${\dot{V}}_{T}$ was low and similar in both groups, indicating that respiratory timing was constant during the CPET and similar in both groups. For the relationship between ${\dot{V}}_{E}$ versus $\dot{V}{\text{CO}}_{2}$, the slope was parallel in the two groups, but with downward shift for the EILO group.

### 4.1 Breathing pattern during exercise in EILO and no‐EILO

The study revealed surprisingly few clear‐cut differences in the breathing patterns between the EILO and no‐EILO groups. Nevertheless, the EILO group was characterized by prolonged inspiratory time, lower inspiratory flow rate, and lower breathing frequency, but similar tidal volume at peak exercise, accompanied by a lower minute ventilation. Lower inspiratory flow rate in patients with EILO appears clearly advantageous in a situation where the narrowest passage in the airway tree (the larynx) becomes compromised in size. FEV_1_, sex, age, and height are all variables of relevance for breathing patterns, and varied between the EILO and no‐EILO groups, but the group differences for breathing pattern remained in the adjusted regression models. We also found that the EILO group had a slightly increased expiratory time, which might be explained by their lower breathing frequency. Walsted et al. assessed some of these variables in six patients with EILO, revealing increased tidal volume and minute ventilation with no difference in breathing frequency or *T*_in_/*T*_tot_ when compared to six subjects without EILO, however, at submaximal work rates and before the onset of laryngeal closure (Walsted et al., 2018). Their finding of higher minute ventilation at submaximal exercise is interesting, as it could indicate hyperventilation and thereby a respiratory alkalosis, which could be involved in causal pathways leading to EILO. Our study does not support this line of reasoning, as minute ventilation was in fact lower at peak exercise. Walsted et al. did not report on these variables at peak exercise, except stating that minute ventilation did “*not differ between the EILO and no*‐*EILO group*,*”* thus, contrasting our finding of a lower minute ventilation.

### 4.2 Breathing pattern in EILO versus no‐EILO described mathematically

The mathematical modeling of the relationships between ${\dot{V}}_{E}$ versus $\dot{V}{\text{CO}}_{2}$ and ${\dot{V}}_{E}$ versus *V*_T_ encompassed data from the complete exercise sessions; that is, from rest to peak exercise. The applied models fitted the data very well in both groups. The quadratic model used to describe the relationship between ${\dot{V}}_{E}$ versus *V*_T_, revealed that five participants who all had EILO had unsatisfactory *R* ^2^ between 0.62 and 0.84, nevertheless, the *p*‐values for all models were highly significant and below 0.001. We studied all these curves closely, searching for features possibly related to the onset of EILO, but the plots did not reflect any abnormalities that were related in time to the onset of laryngeal obstruction as visualized from the CLE test. We also closely examined the relationship *T*_in_/*T*_tot_ over the full range of ${\dot{V}}_{T}$ during the CLE test. This was based on a clinical impression suggesting that the relation *T*_in_/*T*_tot_ changes after developing EILO. However, the coefficient of variation in *T*_in_/*T*_tot_ during the CPET was approximately 12% in both the EILO and no‐EILO groups, indicating that the respiratory timing was similar and approximately constant during incremental exercise.

The linear model applied to mathematically describe the relationship between ${\dot{V}}_{E}$ versus $\dot{V}{\text{CO}}_{2}$ fitted the data very well in both groups. The slopes were similar in the EILO and no‐EILO groups, suggesting a similar breathing pattern throughout the test. However, the intercept related to EILO was lower and significantly related to a higher *T*_in_. Thus, adjusted for *T*_in_, the group with EILO had lower ${\dot{V}}_{E}$ at any given volume of CO_2_, and a higher $\dot{V}{\text{CO}}_{2}$ at a given${\dot{V}}_{E}$. The significance of this finding is uncertain. It could be due to few measuring points after the onset of EILO in some patients. Firm conclusions on this would require blood gas analyses, and the hypothesis to test would be if patients with EILO exercise at a higher level of arterial CO_2_.

Although inspiratory time was prolonged and the breathing frequency and ${\dot{V}}_{E}$ were lower, EILO did not seem to distort the relations between CO_2_ output and ventilation at any point during the exercise session, challenging the notion that hyperventilation is involved in the causal chain leading to EILO or is a consequence of EILO.

### 4.3 Further perspectives—Tools to simplify diagnostic work‐up for EILO

A major aim of this study was the search for less resource demanding tools to diagnose EILO. Given the pivotal role of the larynx in modulating total airway resistance, it appears reasonable to assume that EILO would influence the breathing patterns obtained from standard CPETs. As these tests are routinely performed at exercise laboratories worldwide, this would immensely simplify work‐up for EILO. Analyses of breathing patterns using mathematical regression models have already contributed to a better understanding of respiratory conditions in patients with COPD (Frisk et al., 2015) and lung disease after extreme prematurity (Hestnes et al., 2017). Despite significant differences between the EILO and no‐EILO groups regarding some parameters, there was extensive overlap in the distribution of most parameters in the EILO and no‐EILO groups except inspiratory flow rate. In this dataset, if cut‐off for inspiratory flow rate at peak exercise was set at 210 L/min, only three subjects without EILO would be included and five with EILO would be excluded. Even if low flow rate may suggest EILO, it was also influenced by sex and FEV_1_, both variables that differed between our groups. Thus, this needs to be further investigated before we can conclude.

### 4.4 Strengths and limitations

The main strength of the study was that all participants were examined by a CLE test to ensure that comparisons of the CPET parameters were in fact performed between subjects with and without a moderate/severe EILO and no‐EILO. Another strength was that the no‐EILO group consisted of highly trained individuals with no respiratory complaints, and thus providing a no‐EILO group at good health with an uncomplicated relationship to breathing at high intensity exercise. The different representation of females in the two groups reflects the uneven sex distribution of EILO described in several studies (Roksund et al., 2009; Sandnes, Andersen, et al., 2019; Sandnes, Hilland, et al., 2019). However, this group difference challenges the interpretation of the results, as males and females differ on a range of features relevant to breathing patterns. We carefully adjusted all analyses for relevant group differences, and as exemplified by Table 2, sex differences could not explain the findings of *T*_in_, *B*_f_, ${\dot{V}}_{\text{in}}$, and ${\dot{V}}_{E}$. As our control group could be seen as “supra‐normal” in terms of level of physical activity and general health, this may actually strengthen the notion that findings were scarce, as one could expect even smaller differences with a less fit control group. Nevertheless, these group differences underline that the findings need to be interpreted with caution.

## 5 CONCLUSION

The group of patients with EILO was characterized by prolonged inspiratory time, lower breathing frequency, lower inspiratory flow rate, and lower minute ventilation at peak exercise when compared to a group with no‐EILO. Commonly applied mathematical models could satisfactorily describe breathing patterns in both groups. Comparison of the curve parameters by adjusted regression models revealed that the relationship ${\dot{V}}_{E}$ versus ${\dot{V}}_{T}$ was similar in the two groups, whereas ${\dot{V}}_{E}$ versus $\dot{V}{\text{CO}}_{2}$ had a similar slope, but a lower intercept, suggesting that patients with EILO have a lower minute ventilation at any given volume of CO_2_ compared to the no‐EILO group. For most parameters, there was extensive overlap between the EILO and no‐EILO groups, complicating diagnostic use of the findings. There was one exception to this; low inspiratory flow rate at peak exercise discriminated relatively well between the EILO and no‐EILO groups. However, this needs further investigation before conclusions can be made.

## CONFLICT OF INTEREST

The authors have no conflict of interest to disclose. Haukeland University Hospital owns parts of US patent No. 11/134551, protecting the commercial rights of the CLE test.

## AUTHOR CONTRIBUTIONS’

Ingvild Grønnevik and Astrid Haugen Lie organized data, carried out the initial analyses, drafted the initial manuscript, and revised the manuscript. Ida Hammer coordinated and performed data collection and revised the manuscript. Bente Frisk conceptualized and designed the study, carried out and revised the analyses, and reviewed and revised the manuscript. Thomas Halvorsen and Maria Vollsæter conceptualized and designed the study, and reviewed and revised the manuscript. Ola Drange Røksund conceptualized and designed the study, designed the data collection instruments, performed data collection, and reviewed and revised the manuscript. Hege H. Clemm conceptualized and designed the study, designed the data collection instruments, coordinated and supervised data collection, organized data, carried out analyses, and reviewed and revised the manuscript. All authors approved the final manuscript as submitted, and agreed to be accountable for all aspects of the work.

## Data Availability

In accordance with the approvals granted for this study by The Regional Committee on Medical Research Ethics and The Norwegian Data Inspectorate, the data files are stored securely and in accordance with the Norwegian Law of Privacy Protection. A subset of the data file with anonymized data can be made available to interested researchers upon reasonable request to Hege Clemm, providing Norwegian privacy legislation and GDPR are respected, and that permission is granted from The Norwegian Data Inspectorate and the data protection officer at Haukeland University Hospital.
